# Prognostic gene expression profiling in esophageal cancer: a systematic review

**DOI:** 10.18632/oncotarget.13328

**Published:** 2016-11-12

**Authors:** Els Visser, Ingrid A. Franken, Lodewijk A.A. Brosens, Jelle P. Ruurda, Richard van Hillegersberg

**Affiliations:** ^1^ Department of Surgery, University Medical Center Utrecht, The Netherlands; ^2^ Department of Pathology, University Medical Center Utrecht, The Netherlands

**Keywords:** esophageal cancer, gene expression profiling, response to chemo(radio)therapy, lymph node metastasis, survival, prognosis

## Abstract

**Background:**

Individual variability in prognosis of esophageal cancer highlights the need for advances in personalized therapy. This systematic review aimed at elucidating the prognostic role of gene expression profiles and at identifying gene signatures to predict clinical outcome.

**Methods:**

A systematic search of the Medline, Embase and the Cochrane library databases (2000-2015) was performed. Articles associating gene expression profiles in patients with esophageal adenocarcinoma or squamous cell carcinoma to survival, response to chemo(radio)therapy and/or lymph node metastasis were identified. Differentially expressed genes and gene signatures were extracted from each study and combined to construct a list of prognostic genes per outcome and histological tumor type.

**Results:**

This review includes a total of 22 studies. Gene expression profiles were related to survival in 9 studies, to response to chemo(radio)therapy in 7 studies, and to lymph node metastasis in 9 studies. The studies proposed many differentially expressed genes. However, the findings were heterogeneous and only 12 (ALDH1A3, ATR, BIN1, CSPG2, DOK1, IFIT1, IFIT3, MAL, PCP4, PHB, SPP1) of the 1.112 reported genes were identified in more than 1 study. Overall, 16 studies reported a prognostic gene signature, which was externally validated in 10 studies.

**Conclusion:**

This systematic review shows heterogeneous findings in associating gene expression with clinical outcome in esophageal cancer. Larger validated studies employing RNA next-generation sequencing are required to establish gene expression profiles to predict clinical outcome and to select optimal personalized therapy.

## INTRODUCTION

Esophageal cancer is the eight most common cancer worldwide, with 450.000 new cases and 400.000 estimated deaths per year. [1, 2] The two main types of esophageal cancer, squamous cell carcinoma (SCC) and adenocarcinoma (AC), differ in pathogenesis, epidemiology, tumor biology, prognosis and treatment strategies. [3, 4]

Multimodality treatment, combining esophagectomy with perioperative chemotherapy or neoadjuvant chemoradiotherapy, has been shown to improve patients’ survival and is therefore the standard treatment for curable esophageal cancer. [5–7] However, due to the aggressive character of the tumor and the lack of effective individualized treatment, the survival remains poor with 5-year survival rates of merely 36-47%. [5–8] Moreover, large individual differences in survival, treatment response and metastasis emphasize the need for more personalized therapy. Existing histopathological terms, such as the pathologic TNM classification, are insufficient to accurately predict these individual differences in outcome and to inform personalized treatment. [9–11]

Evidence for the potential prognostic role of gene expression profiles is accumulating. Gene signatures may find clinical application in predicting survival, response to neoadjuvant treatment and metastatic potential. This would enable individualized targeted therapy in order to avoid unnecessary treatment and improve quality of life and longevity.

Prognostic pretreatment gene expression profiles have already been identified through genome-wide microarray analysis for rectal adenocarcinoma [12, 13] and breast cancer [14–16]. With regard to esophageal cancer, multiple studies have suggested a clear association between gene expression and clinical outcome. This systematic review aims to provide an overview of the results, in order to outline the current understanding of the predictive potential of gene expression for survival, response to chemo(radio)therapy, and lymph node metastasis.

## RESULTS

### Study selection and study characteristics

**Figure 1:**
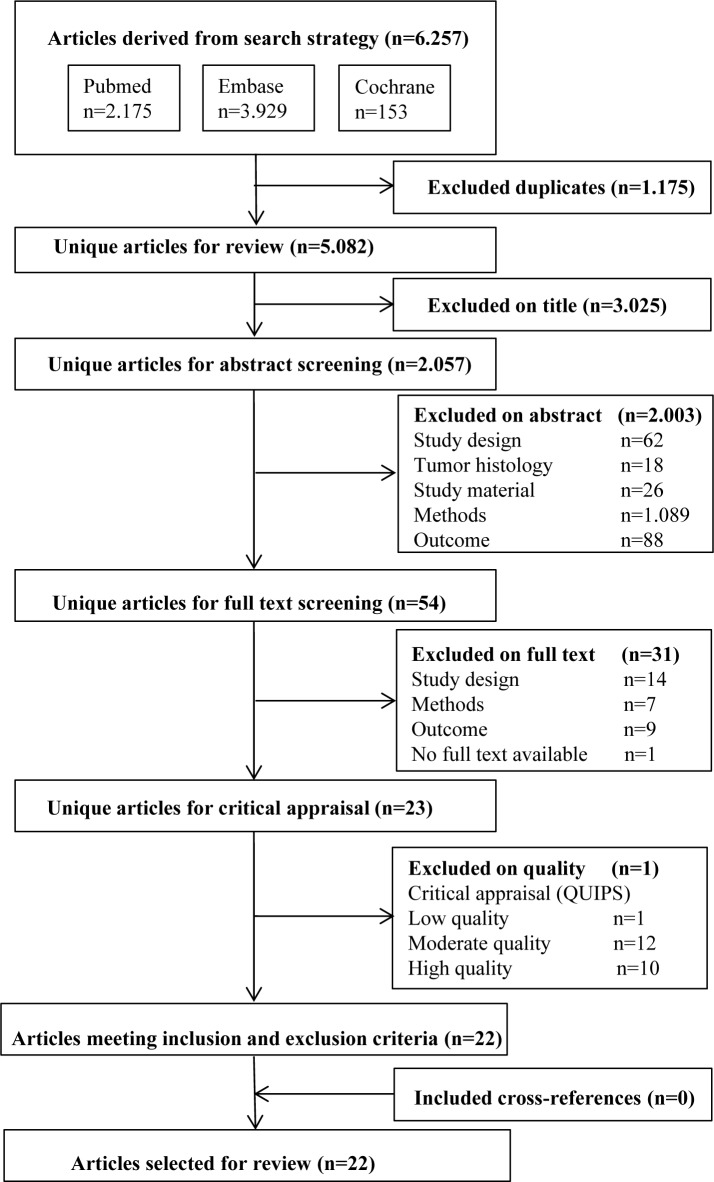
Flowchart search, selection of papers on prognostic gene expression profiling in esophageal cancer (2000-2015)

A flowchart of the systematic literature search and study selection is shown in Figure 1. The systematic search yielded 5.082 unique studies, of which 54 were retrieved for full-text screening. Of these, 23 studies were included according to the criteria (Figure 1) and underwent critical appraisal, using the QUIPS tool (Table 1). One study was excluded as a low quality study [18], while the remaining 12 studies of moderate quality [19–30] and 10 studies of high quality [31–40] were deemed eligible for this review. The 22 eligible studies together included 827 patients, were conducted in 8 countries, and were published between 2003 and 2014. All studies conducted microarray analysis of gene expression profiles, with the exception of 1 study [40] employing RNA next-generation sequencing to analyze transcriptional profiles. When separated on the basis of outcome, 9 studies investigated gene expression profiles in relation to survival [19, 25, 27, 29, 30, 36–38, 40], 7 studies in relation to response to chemo(radio)therapy [24, 27, 28, 32, 34, 35, 39] and 9 studies in relation to lymph node metastasis [20–23, 25, 26, 31, 33, 36].

**Table 1:** Critical Appraisal of reported bias in 6 domains and overall quality according to the Quality in Prognostic Studies Tool

Study	Study participation	Study attrition	Prognostic factor measurement	Outcome measurement	Study confounding	Statistical analysis and reporting	Quality
Ishibashi [19]	+	+	+/−	+	+/−	+/−	Moderate
Kan [20]	+/−	+	+/−	+	+/−	+/−	Moderate
Tamoto [31]	+/−	+	+	+	+/−	+	High
Sato [21]	+/−	+	+	+	+/−	+/−	Moderate
Luthra [32]	+/−	+	+	+/−	+	+	High
Yamabuki [22]	+/−	+	+	-	+/−	+	Moderate
Li [18]	-	+	+/−	+/−	-	+/−	Low
Uchikado [23]	+/−	+	+	+	-	+/−	Moderate
Duong [24]	+/−	+	+/−	+/−	+	+	Moderate
Hammoud [25]	+/−	+/−	+/−	+	+/−	+/−	Moderate
Lagarde [33]	+	+	+	+	+	+	High
Sano [26]	-	+	+/−	+	-	+/−	Moderate
Maher [34]	+	+	+	+	+	+	High
Schauer [35]	+	+	+	+	+/−	+/−	High
Peters[36]	+	+	+	+	+	+	High
Rao [27]	+/−	+	+	+/−	-	+/−	Moderate
Motoori [28]	+/−	+	+	+/−	+/−	+/−	Moderate
Kim [29]	+/−	+	+	+/−	+/−	+/−	Moderate
Goh [30]	+/−	*+*	+/−	+/−	+/−	+/−	Moderate
Pennathur [37]	+	+	+	+	+	+	High
Lu [38]	+/−	+	+	+/−	+	+	High
Wen [39]	+	+	+	+	+	+	High
Lin [40]	+	+	+	+/−	+	+/−	High

+ low bias, +/− moderate bias, - high bias

### Gene expression and survival

The 9 studies [19, 25, 27, 29, 30, 36–38, 40] that studied gene expression in association with survival are summarized in Table 2. In 7 studies, patients underwent esophagectomy only and resection specimens were obtained as study material, either fresh frozen [19, 27, 30, 36–38, 40] or formalin-fixed paraffin-embedded (FFPE) [25]. In 2 studies, esophagectomy was preceded by chemotherapy [27] or chemoradiotherapy [29] and pretreatment biopsies were obtained as study material. Prognostic genes were identified through correlation to continuous survival [25, 36, 38], comparison between a poor and good survival group [27, 40] or used to form patient clusters [19, 29, 30, 37]. Genes with differential expression between tumor and normal tissue [30, 38] were not regarded as prognostic genes. After gene expression profiling, 6 studies [27, 29, 30, 36–38] proceeded with the identification of a prognostic gene signature, which was validated in an external cohort in 4 studies [29, 30, 36, 38].

**Table 2:** Articles identifying genes associated with survival in esophageal AC and SCC (2000-2015)

Study (year)	Country	*n*	Histological tumor type	Survival assessment	Median follow-up/ overall survival	Methods	Prognostic genes (cut-off point)	Gene signature	Signature validation (*n* samples)
Hammoud (2009) [25]	USA	89	AC	Continuous	FU 25 months (range 2-132) OS 2.18 years	DASL 502 cancer genes	9 genes (continuous)	Not identified	Not conducted
Kim (2010) [29]	USA	64	AC	3 clusters; Poor survival cluster; mean OS< 13 months	Not reported	48 K Oligo-nucleotide microarray	452 (1.5-fold), 10 genes (4-fold)	2 genes	10 genes (52)
Peters (2010) [36]	UK	75	AC	Continuous	FU 20 months (range 0.5-137) FU survivors 89 months (range 66-137)	44 K Oligo-nucleotide microarray	119 genes (continuous), 10 genes (1.5-fold)	4 genes	4 genes (371)
Goh (2011) [30]	UK	56	AC	5 clusters; Poor survival cluster; median OS 1.37 years Remaining clusters; median OS 2.74 years	FU survivors (min. 5years)	44 K Oligo-nucleotide microarray	Not identified	4 genes	4 genes (371)
Rao (2011) [27]	UK	35	AC	Poor survival group; OS <570 dys Good survival group; OS >570dys survival	FU 938 days (min. 500) OS 570 days	22 K cDNA microarray	165 genes (1.4-fold)	165 genes	Not conducted
Pennathur (2013) [37]	USA	64	AC	2 clusters; Poor survival cluster; median OS 19 months (95%CI 10-25) Good survival cluster; Median OS not reached	OS 27 months (95%CI lower limit 22) FU survivors 34 months (range 2-76)	Affymetrix U133 microarray	59 genes (2-fold)	59 genes	Not conducted
Ishibashi (2003) [19]	Japan	12	SCC	2 clusters; Recurrence cluster; median DFS 280 dys Non-recurrence cluster; median FU 483 dys	OS (range 121-644 days)	Affymetrix 12,6 K microarray	multiple genes (continuous)	Not reported	Not conducted
Lu (2014) [38]	China	10	SCC	Continuous	Not reported	Affymetrix U133 microarray	Not identified	1 gene	1 gene (198)
Lin (2014) [40]	China	8	SCC	Recurrence group; Recurrence and death <2yrs Non-recurrence group; DFS ≥5yrs	Not reported	Ion Total RNA-Seq Kit v2	533 genes (2-fold)	Not identified	Not conducted

n = number, AC = adenocarcinoma, SCC = squamous cell carcinoma, FU = follow-up, OS = overall survival, DFS = disease-free survival

#### Adenocarcinoma

Of the 6 studies [25, 27, 29, 30, 36, 37] conducted on AC, 5 studies [27, 29, 30, 36, 37] reported gene signatures associated with survival. One study [36] reported and validated a prognostic 4-gene signature (and showed that underexpression of DCK, PAPSS2 and SIRT2 in combination with overexpression of TRIM44 decreased 5-year survival from 58% to 14%. A second study [29] discovered clusters of patients with differential gene expression profiles and further investigated genes with overexpression in the poor prognosis cluster. External validation of these genes showed a significant association between a 2-gene signature, with combined overexpression of SPARC and SPP1, and poor survival. A third study [30] also performed cluster analysis and found that another validated 4-gene signature (EGFR, MTMR9, NEIL2, and WT1) was able to stratify patients into 5 survival clusters. A similar study identified a 165-gene [37] signature to classify patients into a good survival cluster and poor survival cluster. The last study [27] took another approach to divide a cohort of patients into a good survival group and a poor survival group and compared gene expression between both groups to find a 59-gene signature predictive of survival.

#### Squamous cell carcinoma

As for SCC, only 1 [38] of the 3 studies [19, 38, 40] identified a prognostic gene signature. This study [38] found an association between overexpression of the randomly selected gene CTTN and shorter disease-free survival in an external validation cohort.

### Gene expression and response to chemo(radio)therapy

The 7 studies [24, 27, 28, 32, 34, 35, 39] that analyzed gene expression in association with response to chemo(radio)therapy are summarized in Table 3. Patients received neoadjuvant chemotherapy in 3 studies [27, 28, 35] and neoadjuvant chemoradiotherapy in 4 studies [24, 32, 34, 39], following varying regimens as specified in Table 2. All studies obtained fresh frozen pretreatment endoscopic biopsies for microarray analysis. Response to chemo(radio)therapy was defined differently in each study and evaluated on the basis of resection specimens in 4 studies [32, 34, 35, 39] and on the basis of (a combination of) medical imaging techniques in 3 studies [24, 27, 28]. All studies proposed a gene signature prognostic for response to chemo(radio)therapy, which was externally validated in 3 studies [28, 34, 39].

**Table 3:** Articles identifying genes associated with response to chemo(radio)therapy in esophageal AC and SCC (2000-2015)

Study (year)	Country	*n*	Histological tumor type	Treatment	Response Evaluation	Response definition (n responders)	Method	Prognostic genes (cut-off point)	Gene signature	Signature validation (*n* samples)
Schauer (2009) [35]	Germany	47	AC	CT (Cisplatin/ 5-FU/Leucoverin) + Surgery	Resection specimen	<50% viable tumor cells (19)	Affymetrix U133 microarray	86 genes (2-fold)	1 gene	Not conducted
Rao (2010) [27]	UK	35	AC	CT (Epirubicin/ Cisplatin/Capecitabine) + Surgery (26)	CT-scan, EUS	RECIST criteria (18)	22 K cDNA microarray	113 genes (1.4-fold) (EUS)	113 genes	Not conducted
Motoori (2010) [28]	Japan	25	SCC	CT (Cisplatin/ 5-FU/ Doxorubicin) + Surgery (17)	CT-scan	<50% viable tumor cells (11)	30K oligo-nucleotide microarray	19.166 genes (continuous)	199 genes	199 genes (10)
Wen (2014) [39]	China	28	SCC	CRT (Cisplatin/ Vinorelbine/40Gy) + Surgery	Resection Specimen	No residual tumor cells (11)	Affymetrix U133 microarray	178 genes (continuous), 10 genes (2-fold)	3 genes	3 genes (32)
Luthra (2005) [32]	USA	19	AC (16) SCC (2) ASCC (1)	CRT (Docetaxel/ 5-FU/ Irinotecan/ 50.4Gy) + Surgery	Resection Specimen	No residual tumor cells (10)	Affymetrix 22 K microarray	80 genes (2-fold)	3 genes	Not conducted
Duong (2007) [24]	Australia	46	AC (25) SCC (21)	CRT (Cisplatin/ 5-FU/ 35-50Gy) + Surgery	CT scan, endoscopy with biopsy, FDG-PET	Metabolic response and no residual tumor (17)	10,5 K cDNA microarray	Not reported	32 genes (SCC)	Not conducted
Maher (2009) [34]	Ireland	13	AC (10) SCC (3)	CRT (Cisplatin/ 5-FU/40.5-44Gy) + Surgery	Resection Specimen	Fibrosis with no (TRG1) or rare (TRG2) residual tumor cells (4)	32 K oligo-nucleotide microarray	103 genes (continuous), 67 genes (2-fold)	12 genes	12 genes (27)

n= number, AC= adenocarcinoma, SCC= squamous cell carcinoma, CT = chemotherapy, CRT = chemoradiotherapy, 5-FU = 5-Fluorouracil, CT-scan = Computerized Tomography-scan, EUS= endoscopic ultrasound, FDG-PET= Fluorodeoxyglucose Positron Emission Tomography, RECIST = Response Evaluation Criteria in Solid Tumors, TRG = Tumor Regression Grade.

#### Adenocarcinoma

Two studies [27, 35] found prognostic genes and a gene signature for AC only. One study [35] compared gene expression between responders and non-responders to chemotherapy. The results demonstrated a significant correlation between the overexpression of Ephrin B3 and response. Another study [27] identified, on the basis of endoscopic ultrasound (EUS) only, a 113-gene signature correlated with chemotherapy response.

#### Squamous cell carcinoma

Two studies [28, 39] were conducted on SCC only. One study [28] documented a 199-gene signature and showed in external validation that 1 gene (PERP) was underexpressed and 4 genes (DAD1, PRDX6, SELPINB6 and SRF) were overexpressed in non-responders compared to responders to chemotherapy. Similarly, another study [39] identified a 3-gene signature and found that a combination of underexpression of ClOrf226 and LIMCHI1, and overexpression of MMP1 was predicitive for responders.

#### Adenocarcinoma and squamous cell carcinoma

Three studies [24, 32, 34] obtained biopsies of both AC and SCC. The first study [34] found differentially expressed genes, of which 12 genes were selected for external validation. A model of 5 genes reported that underexpression of 4 genes (EPB41L3, NMES1, RNPC1, STAT5B) and overexpression of 1 gene (RTKN), was able to identify responders from non-responders to chemoradiotherapy. The second study [32] showed that overexpression of 3 genes (PERP, S100A2 and SPRR3) was able to characterize complete responders to chemoradiotherapy. The third study [24] investigated differential expression in both AC and SCC and identified a 32-gene signature that was predictive for response to chemoradiotherapy in SCC only.

### Gene expression and lymph node metastasis

Table 4 summarizes the 9 articles [20–23, 25, 26, 31, 33, 36] that investigated gene expression profiles in relation to lymph node metastasis. All patients underwent esophagectomy only and fresh frozen resection specimens were obtained as study material. In 6 studies [22, 23, 25, 26, 31, 36] the (International Union Against Cancer (UICC)) TNM classification was used by pathologists to assess lymph node metastasis and in 3 studies [20, 21,33] no form of classification was stated. All 9 studies identified prognostic genes with differential expression between patients groups with node-negative (N0) *versus* node-positive (N+) tumors. Of these, 4 studies [20, 22, 26, 31] reported a gene signature, which was validated in an external cohort in 3 studies [20, 26, 31].

**Table 4:** Articles identifying genes associated with lymph node metastasis in esophageal AC and SCC (2000-2015)

Study (year)	Country	*n*	Histological tumor type	Classification for lymph node assessment	Compared N stage (n samples)	Method	Prognostic genes (cut-off point)	Gene signature	Signature validation (*n* samples)
Lagarde (2008) [33]	The Netherlands	61	AC	Not reported	N0 (17) N+ (44)	44 K oligonucleotide microarray	5 genes (continuous)	Not reported	Not conducted
Hammoud (2008) [25]	USA	89	AC	TNM classification	N0 (23) N+ (66)	DASL 502 cancer genes	17 genes (continuous)	Not identified	Not conducted
Peters (2010) [36]	UK	75	AC	TNM classification	N0 (14) N+ (48)	44 K Oligonucleotide microarray	270 genes (1.5-fold)	Not identified	Not conducted
Tamoto (2004) [31]	Japan	36	SCC	UICC TNM classification	N0 (16) N+ (20)	1,3 K cDNA microarray	87 genes (1.2-fold)	44 genes	44 genes (18)
Kan (2004) [20]	Japan	15	SCC	Not reported	N0 (6) N+ (9)	8,1 K cDNA microarray	120 genes (continuous)	58 genes	58 genes (13)
Sato (2006) [21]	Japan	54	SCC	Not reported	T1N0 (9) T1N+ (10), T2-4N0 (7) T2-4N+ (28)	Affymetrix 22 K microarray	78 genes (continuous)	Not identified	Not conducted
Yamabuki (2006) [22]	Japan	19	SCC	UICC TNM classification	N0 (6) N+ (13)	32 K cDNA microarray	34 genes (5-fold)	20 genes	Not conducted
Uchikado (2006) [23]	Japan	16	SCC	UICC TNM classification	N0 (5) N+ (11)	17 K oligonucleotide microarray	181 genes (not reported)	Not identified	Not conducted
Sano (2009) [26]	Japan	35	SCC	UICC TNM classification	N0 (11), >5 positive nodes (25)	Affymetrix U95A microarray	209 (continuous)	6 genes	6 genes (66)

n = number, AC = adenocarcinoma, SCC = squamous cell carcinoma, UICC = International Union Against Cancer, N = number of lymph nodes, according to TNM-6 classification.

#### Adenocarcinoma

In AC, 3 studies [25, 33, 36] identified differentially expressed genes but none reported a gene signature to predict N+ tumors.

#### Squamous cell carcinoma

In SCC, 6 studies [20–23, 26, 31] documented genes with differential expression and 4 studies [20, 22, 26, 31] developed a gene signature predictive for lymph node involvement. Three studies [20, 22, 26, 31] compared gene expression between N0 tumors and N+ tumors and identified a gene signature consisting of 20 [22], 44 [31] and 58 [20] genes, of which the latter 2 were externally validated. Another study [26] compared gene expression between node-negative patients and patients with more than 5 metastatic lymph nodes. This study further reported and validated a gene signature of 6 genes. Overexpression of CALB1, CEA/CEACAM5, CLDN10, KRT7/CK7, MUC1, and TFF3 was associated with more than 5 metastatic lymph nodes.

### Overlap in gene expression

Supplementary Table S3 combines the available results of all reviewed studies into a list of genes for each of the 3 outcomes per histological tumor type. In association with survival, the 9 studies identified a total of 1.337 genes (excluding 1 study [19] that did not specify the number of multiple genes identified with differential expression), of which 277 genes (21%) (range 1-113 per study) were reported. Only for AC, overlap between the studies was found with respect to 9 genes (ALDH1A3, BIN1, CSPG2, DOK1, IFIT1, IFIT3, PHB, SPP1) which were described by 2 of the 5 studies. For response to chemo(radio)therapy, the 7 studies identified a total of 19.726 genes (excluding 1 study [28] that identified 19.166 genes with differential expression), of which 158 genes (1%) (range 1-49 per study) were reported. No overlap in genes was found between the studies. The 9 studies that studied gene expression in relation with lymph node metastasis identified a total of 1.001 genes and reported a total of 677 genes (68%) (range 5-252 per study). Of these, 3 genes (ATR, MAL, PCP4) were described in 2 studies investigating SCC.

## DISCUSSION

This systematic review aimed to identify prognostic genetic profiles in esophageal carcinoma. The results demonstrate a large heterogeneity in gene expression profiles and gene signatures predicting survival, response to chemo(radio)therapy, and lymph node metastasis. None of the identified gene signatures is directly applicable in clinical practice at present. However, microarray analysis and genome sequencing might provide valuable information for predicting individual variations in clinical outcome and establishing personalized therapy for esophageal cancer patients in the near future.

A careful literature search and quality assessment were directed at including all studies of moderate and high quality relevant to prognostic gene expression. The 22 included studies were assessed on the same criteria and genes with prognostic relevance were retrieved from the article or through additional contact with study authors if possible. In support of a prognostic role, all studies, except 2 [30, 38], identified genes with differential expression with regard to the investigated outcome and 16 studies [20, 22, 24, 26–32, 34–39] identified and reported a gene signature. In consistence with the distinct epidemiology and tumor biology of AC and SCC [4, 11], most studies conducted in East Asia included exclusively SCC and most studies in western countries focused on AC only. Of the 3 studies [24, 32, 34] investigating both AC and SCC, 2 studies [32, 34] described a gene signature for both tumor types, while the third study [24] found a gene signature that was predictive for SCC only.

Despite the thorough review, studies to current date have been heterogeneous in both methods and results. Therefore, comparison of the different studies has been unable to establish a repeatedly identified gene signature with clinical relevance. Studies differed largely in the documented number of prognostic genes and genes were not reported nor provided after contacting authors in 8 articles [26–28, 32, 35, 37, 39, 40]. Although the 22 studies identified a large number of prognostic genes (Tables 2, 3 and 4), only 1.112 genes were reported and included in Supplementary Table S3. Comparison of data per outcome and histological subtype showed that only a 12 genes return in more than 1 study, suggesting a high false positive rate of identified genes. Only 9 genes (ALDH1A3, BIN1, CSPG2, DOK1, IFIT1, IFIT3, PHB, SPP1) were described in 2 studies on survival in AC and only 3 genes (ATR, MAL, PCP4) on lymph node metastasis in SCC. There was no overlap between studies investigating response to chemo(radio)therapy.

The heterogeneity in identified genes can be attributed to several factors. Firstly, the studies used limited sample sizes and thus individual genetic variability may have largely impacted the identified genes. The studies included 8 to 89 patients, with only 3 studies [25, 33, 36] investigating 75 or more patients. Moreover, only 10 [20, 26, 28–31, 34, 36, 38, 39] of the 16 reported gene signatures were validated in external cohorts. Validation in independent cohorts increases reliability of the gene signatures and is required to achieve clinical implementation. In addition, the included studies showed large methodological variation in treatment of patients, definition and evaluation of outcome, employed microarray analysis and chosen cut-off point for differential expression.

The studies investigating survival showed heterogeneity in whether gene expression was correlated to continuous survival [25, 36, 38], compared between differently defined poor and good survival groups [27, 40] or used to create patient clusters [19, 29, 30, 37]. More importantly, response to chemo(radio)therapy was assessed using computerized tomography (CT) scans, Fluorodeoxyglucose Positron Emission Tomography (FDG-PET), or endoscopic ultrasound (EUS) with or without biopsy in 3 studies [24, 27, 28]. Accuracy of these imaging techniques in evaluating response is suboptimal compared to histopathology, which is the gold standard [41–43]. Conclusions on response to chemo(radio)therapy were further complicated by the use of different chemotherapy regimens and varying definition of response. Response was defined as either 50% reduction of viable tumor cells [28, 35] or absence of residual tumor cells [24, 32, 34, 39]. These are 2 distinct predictive categories of partial response and complete response, respectively. [44, 45] Similarly, the studies investigating lymph node metastasis differed in the classification system used and the lymph node-stage compared.

Despite these limitations, the current findings show that gene signatures can be of great prognostic value for clinical outcomes and are therefore paramount in understanding pathogenesis and selecting optimal personalized therapy for the individual patient. A gene signature to predict survival may be able to explain why some patients with good tumor characteristics show shorter disease-free survival than expected, and vice versa, thus offering information that is not accurately provided by the pathologic TNM classification. [46, 47] Moreover, patients who are unlikely to benefit from chemo(radio)therapy could be selected to receive direct surgical resection, avoiding unnecessary toxicity [7, 48] and delay in surgical treatment with risk of disease progression. Conversely, a restrictive and non-surgical approach with less comorbidity might be considered for patients who are likely to be complete responders to chemo(radio)therapy. In addition, the identification of a gene signature to predict lymph node metastasis would be a powerful diagnostic tool. Lymph node-negative patients could receive limited-field radiotherapy with reduced treatment-related toxicities. [49] Furthermore, an invasive extended lymphadenectomy might be avoided in these patients, limiting the risk of postoperative morbidity. [50] Although the included studies did not investigate other prognostic variables as tumor differentiation, perineural and angioinvasive growth, gene signatures for these variables can be of great value in the future as well.

This systematic review shows potential for prognostic gene expression analysis and future research should aim at translation to clinical practice. An international consortium dedicated to large-scale data sharing and using a clear methodological ‘gold standard’ to perform analyses can resolve inconsistencies among the reported gene expression profiles. [51] In addition, future studies can yield more reliable results by conducting gene expression profiling on larger samples and by validating signatures in independent patient cohorts. This would allow for the development of a gene signature with direct clinical relevance, similar to the 70-gene signature ‘MammaPrint’ to predict survival of patients with breast cancer [14–16] and the 42-gene ‘ColoPrint’ to predict disease relapse in early stage colon cancer [12, 13]. In addition to microarrays analysis, 1 study [40] employed RNA next-generation sequencing. This exciting emerging technique can provide additional information on gene fusions and alternative splicing with high accuracy and sensitivity [52, 53]. Advances in microarray analysis and next-generation sequencing will form valuable tools to realize personalized medicine for patients with esophageal cancer in the near future.

## MATERIALS AND METHODS

### Search strategy

A systematic literature search was conducted of the Medline (*via* PubMed), Embase and Cochrane library databases, using the limits ‘human’, ‘English language’ and ‘publication date 2000-2015′. The medical subject headings (MeSH) and their synonyms concern ‘esophageal neoplasm’, in combination with ‘DNA/RNA sequence analysis’ or ‘gene expression’, in relation to ‘response to chemo(radio)therapy’, ‘metastasis’, ‘survival’, ‘prognosis’ or ‘recurrence’. The complete search strategy is provided in the supplementary Table S1. The search was last updated on June 30 2016.

### Study selection

Studies identified by the search strategy were evaluated for eligibility by independent dual author review (EV and IF). Discrepancies between the two reviewers were resolved by consensus. After removal of duplicates, studies that seemed unrelated to the study aims were excluded in title screening. The remaining articles underwent subsequent abstract and full text screening, based on carefully constructed inclusion and exclusion criteria (Supplementary Table S2). These criteria aimed at inclusion of original studies, that conducted microarray analysis or genome sequencing on untreated cancer biopsies or resection specimens of AC or SCC, and associated the genetic profile to survival, response to chemo(radio)therapy and/or lymph node metastasis. A manual cross-reference search was performed in the reference lists of the eligible articles to assure that relevant related articles were included in this study.

### Quality assessment

Studies eligible on the basis of the inclusion and exclusion criteria were subsequently evaluated in a critical appraisal, using the Quality in Prognostic Studies (QUIPS) tool. [17] The quality of studies was assessed on the basis of bias in study participation, study attrition, prognostic factor measurement, outcome measurement, study confounding and statistical analysis plus reporting. Studies were assigned low, moderate or high risk of bias in each of these 6 domains. In case of discrepancies in quality assessment between the two authors (EV and IF), a consensus was reached through discussion. The overall quality of each study was scored using a three-point scale (low, moderate and high quality). Low quality studies, defined as high bias in at least 2 of the 6 domains, were excluded from this study. Studies were defined to be of high quality if they scored low bias on at least 4 domains in the absence of any high bias score. Both high quality studies and the remaining studies of moderate quality were included in this study.

### Data extraction

Data were collected independently by two authors (EV and IF). The following information was obtained from the included studies: first author's name, publication year, country of origin, sample size, histological tumor type, treatment, study material, definition of outcome, sequencing method or microarray analysis, cutoff value for expression, method of analysis, identified prognostic genes and/or gene signature, and validation. Gene signatures consisting of less than 10 genes were mentioned in the text. If data on any of the above items were not reported in the study, items were indicated as “not reported”. Authors were contacted for important information that was missing or unclear.

### Data presentation

The above-mentioned data were presented per study in tables, making a distinction between survival, response to chemo(radio)therapy and metastasis and separating AC and SCC. Studies were compared on the basis of treatment and study material as well as methods used to identify genes, such as chosen microarray analysis, and validation. When available, the identified prognostic genes and gene signatures were described. A list of prognostic genes was constructed, combining the results of all studies per outcome and histological tumor type. Genes reported in more than 1 study were highlighted. When studies investigated differential gene expression on multiple cut-off points, genes identified by the lowest cut-off point were included in the list of genes.

## SUPPLEMENTARY MATERIAL TABLES


