# Supplementary material for: Prognostic gene expression profiling in esophageal cancer: a systematic review
Source: Oncotarget. 2016 Nov 12;8(3):5566–77. doi: 10.18632/oncotarget.13328 (PMC5354930; doi:10.18632/oncotarget.13328)
Supplement: Supplementary file 1 [file oncotarget-08-5566-s001.pdf]

Prognostic gene expression profiling in esophageal cancer - a systematic review

Supplementary Material

Supplementary Table S1. Full text of search strategy results as of June 30, 2016

No.	Search query	PubMed	Embase	Cochrane
1	Domain: (((Esophageal OR Oesophageal)) AND (Cancer OR Cancers OR Carcinoma OR Carcinoma[MeSH Terms] OR Carcinomas OR Neoplasm OR Neoplasm[MeSH Terms] OR Neoplasms)) OR Esophageal Neoplasm[MeSH Terms])	24.373	85.397	2.455
2	Determinant: ((Sequence Analysis OR Sequence Analysis[MeSH Terms] OR Sequence Analysis, DNA OR Sequence Analysis, DNA[MeSH Terms] OR Sequence Analysis, RNA OR Sequence Analysis, RNA[MeSH Terms]) OR ((Gene OR Genes OR Genes[MeSH Terms] OR Genome OR Genome[MeSH Terms] OR Exome OR Exome[MeSH Terms] OR DNA OR DNA[MeSH Terms] OR RNA OR RNA[MeSH Terms]) AND (sequence OR sequencing OR analysis OR analyse OR express OR expression)))	859.682	3.010.057	20.890
3	Outcome: ((Survival OR Survival[MeSH Terms] OR Prognosis OR Prognosis[Mesh Terms] OR Recurrence OR Recurrence[Mesh Terms] OR Recurrent Disease OR Neoplasm Recurrence, Local OR Neoplasm Recurrence, Local[MeSH Terms]) OR ((Response AND (Neoadjuvant Therapy OR Neoadjuvant Therapy[Mesh Terms] OR Chemoradiotherapy OR Chemoradiotherapy[Mesh Terms] OR Chemoradiation OR Chemotherapy)) OR ((Radiosensitivity) OR Chemosensitivity)) OR (Neoplasm Metastasis OR Neoplasm Metastasis[MeSH Terms] OR Lymphatic Metastasis OR Lymphatic Metastasis[MeSH Terms]))	1.535.527	1.658.610	1.169
4	#1 AND #2 AND #3	2.175	3.929	153

Filters: publication date from 2000/01/01 to 2015/12/31, humans, English

Supplementary Table S2. Inclusion and Exclusion criteria

	**Inclusion**	**Exclusion**
**Study design**	Original study<br>General patient population undergoing esophagectomy	Review<br>Meta-analysis<br>Case study
**Histological tumor type**	Adenocarcinoma<br>Squamous cell carcinoma	Barrett's lesion<br>Small cell esophageal cancer<br>HPV-related esophageal cancer
**Study material**	Untreated esophageal cancer biopsies<br>Untreated esophageal cancer resection specimens	Cell-lines<br>Serum<br>Metastases
**Methods**	Microarray analysis<br>Whole exome/genome sequencing	Immunohistochemical staining<br>Real-time Polymerase Chain Reaction<br>Tissue Microarray<br>Proteomics<br>MicroRNA analysis<br>Copy number alterations
**Outcome**	Response to chemo(radio)therapy<br>Lymph node metastasis<br>Recurrence, prognosis, overall survival	Distinction between histological subtypes<br>Response to adjuvant therapy<br>Response to radiotherapy
